# Supplementary material for: Recommendations for the Clinical Approach to Immune Thrombocytopenia: Spanish ITP Working Group (GEPTI)
Source: J Clin Med. 2023 Oct 10;12(20):6422. doi: 10.3390/jcm12206422 (PMC10607106; doi:10.3390/jcm12206422)
Supplement: Supplementary file 1 [file jcm-12-06422-s001.zip › jcm-2621147-supplementary.pdf]

Supplementary Table S1. Immunomodulators, immunosuppressants and cytostatic agents for the rescue of patients with multirefractory primary ITP

Drug	Dose	Route	Response after 1 month (%)	Sustained response (%)	Side effects
Azathioprine	50-300 mg/day	Oral	–	51-64	Neutropenia, nausea, infection, liver toxicity
Cyclosporine	2.5-6 mg/kg/day	Oral	38-57	23-44	Asthenia, weakness, AHT, gingival hyperplasia, neuropathy, renal toxicity
Mycophenolate mofetil	250-1,000 mg, twice daily	Oral	–	57-62	Diarrhea, cephalea
Danazol	400-800 mg/day	Oral	24-58	9.5-96	Weight gain, hair loss, amenorrhea, virilization, liver toxicity
Dapsone	50-100 mg/day	Oral	36-63	0-55	Hemolytic anemia, methemoglobinemia, skin affection
Cyclophosphamide	1-2 mg/kg/day	Oral	10-70	60	Myelosuppression, infection, secondary neoplasm, infertility, hemorrhagic cystitis
Vinca alkaloids	1-2 mg/wk VC; 5-10 mg/wk VB	i.v.	–	0-42	Myelosuppression, neuropathy, constipation

AHT, arterial hypertension; ITP, immune thrombocytopenia; i.v., intravenous; VB, vinblastine; VC, vincristine; wk, week.

Supplementary Table S2. A proposal for the progressive dose reduction and suspension of treatment with TPO-RA

**Dose de-escalation proposal**
Eltrombopag
Reduce 30% dose in each step
Monitor patient each 2 weeks: with stable platelet counts >50x10^9/L, introduce a new dose-reduction step each 2-4 weeks
Romiplostim
Reduce 1mg/kg/week in each step
Avatrombopag
Reduce dose progressively according to technical sheet
**Suitability** [73,74,76]
Candidate patients
Patients with platelet counts >100×10^9/L (complete response) which have been maintained for ≥6 months without rescue treatment
Patients with platelet counts >50×10^9/L in 75% of controls during ≥6 months
Non-indicated patients
Patients with history of severe or life-threatening bleeding and poor response to rescue therapy
Patients with history of unsteady platelet count values
Patients with comorbidities increasing hemorrhagic risk
Patients in treatment with anticoagulant or antiplatelet agents
Patients with history of sharp drop of platelet count values after a slight dose modification
Patients with platelet count values in the range of 30-50x10^9/L

TPO-RA, agonist of thrombopoietin receptor.
